# Identification of Novel Melanin Synthesis Inhibitors From *Crataegus pycnoloba* Using an *in Vivo* Zebrafish Phenotypic Assay

**DOI:** 10.3389/fphar.2018.00265

**Published:** 2018-03-26

**Authors:** Adamantia Agalou, Michael Thrapsianiotis, Apostolis Angelis, Athanasios Papakyriakou, Alexios-Leandros Skaltsounis, Nektarios Aligiannis, Dimitris Beis

**Affiliations:** ^1^Zebrafish Disease Model Lab, Biomedical Research Foundation Academy of Athens, Athens, Greece; ^2^Department of Pharmacognosy and Natural Product Chemistry, Faculty of Pharmacy, University of Athens, Athens, Greece; ^3^Institute of Biosciences and Applications, National Centre of Scientific Research “Demokritos”, Athens, Greece

**Keywords:** phenotype-driven screens, zebrafish, *Crataegus*, melanin synthesis inhibitors, dibenzofuran

## Abstract

Zebrafish has emerged as a powerful model organism for high throughput drug screening. Several morphological criteria, transgenic lines and *in situ* expression screens have been developed to identify novel bioactive compounds and their mechanism of action. Here, we used the inhibition of melanogenesis during early zebrafish embryo development to identify natural compounds that block melanogenesis. We identified an extract from the Greek hawthorn *Crataegus pycnoloba* as a potent inhibitor of melanin synthesis and used activity based subfractionation to identify active subfractions and eventually three single compounds of the same family (dibenzofurans). These compounds show reversible inhibition of melanin synthesis and do not act via inhibition of tyrosinase. We also showed that they do not interfere with neural crest differentiation or migration. We identified via *in silico* modeling that the compounds can bind to the aryl hydrocarbon receptor (AHR) and verified activation of the Ahr signaling pathway showing the induction of the expression of target genes.

## Introduction

Melanin is synthesized in melanosomes, special organelles within the melanocytes, via a process called melanogenesis. The amount, the type and the distribution pattern of melanin determine the actual skin, hair and eye color of humans and animals (Park et al., 2009). Melanogenesis is a complex metabolic pathway that combines enzymatically catalyzed and spontaneous chemical reactions. It is initiated by the hydroxylation of tyrosine to L-DOPA (3,4-dihydroxyphenylalanine), which further undergoes oxidation to dopaquinone. Both of these reactions are catalyzed by the key enzyme tyrosinase (TYR) and comprise the rate limiting steps in melanin synthesis, since the rest of the synthesis can proceed spontaneously at physiological pH. After dopaquinone production, two main pathways diversify, leading in the end to the synthesis of two types of melanin, the black–brown eumelanin and the yellow–red pheomelanin (Chang, 2012; Speeckaert et al., 2014).

During embryo development, melanocytes initially derive from the neural crest. They differentiate from a multipotential stem cell population and migrate throughout the embryo to specific sites in the body (Mort et al., 2015). Melanocyte differentiation requires the cross-talk of several signaling pathways, including Wnt, Notch, and BMP, and is characterized by the expression of specific genes in each step of melanocyte lineage development. For example, SOX10 is an early marker of specified ectomesenchymal neural crest cells from which the pigment lineage will arise, while the receptor TYR kinase KIT is required for migration and proliferation of the embryonic melanocyte progenitors. Microphthalmia-associated transcription factor (MITF), a downstream target of SOX10, promotes melanocyte differentiation from the neural crest (Hou et al., 2006). MITF is the master regulator of melanocyte identity and transcriptionally activates several genes involved in melanin synthesis including TYR and dopachrome tautomerase (DCT) (White and Zon, 2008; Yamaguchi and Hearing, 2009).

Melanin has various important physiological functions, including protection of the skin from ultraviolet (UV) damage, inhibition of photocarcinogenesis (Kadekaro et al., 2005; Costin and Hearing, 2007), removal of reactive oxygen species (ROS) (Herrling et al., 2008) and the synthesis of vitamin D (Shoenfeld et al., 2009). However, excessive synthesis of melanin and its accumulation in the skin can cause various pigmentation disorders such as melasma, post-inflammatory hyperpigmentation (Tsatmali et al., 2002; Briganti et al., 2003; Hearing, 2011), and melanoma (Liu et al., 2014; Mort et al., 2015).

Melanoma, a cancer originating from melanocytes, is the most aggressive, metastatic and lethal form of skin cancer. The steps by which a normal melanocyte becomes or generates the melanoma are largely unknown (Ceol et al., 2008). Recent studies revealed that the reemergence of progenitor identity might be a rate-limiting step in the initiation of metastatic melanoma. Kaufman et al. (2016) reported the development of an elegant zebrafish transgenic reporter system that allows the tracking of the early steps of tumor initiation *in vivo*. They found that oncogene-expressing melanocytes are dedifferentiating and reprogrammed into neural crest-like progenitors before they progress into invasive tumor-cells.

Several studies have focused on inhibition of melanogenesis and the prevention of abnormal pigmentation for medical and cosmetic benefits (Solano et al., 2006; Gillbro and Olsson, 2011). Strategies for searching of melanogenesis inhibitors include *in silico, in vitro*, and *in vivo* approaches (Solano et al., 2006; Chang, 2012; Khan, 2012; Chen et al., 2015; Hassan et al., 2016). A number of melanogenesis inhibitors are currently being utilized as pharmaceutical or cosmetic additives, however, due to their adverse side effects (skin irritation, cytotoxicity, carcinogenicity), the low formulation stability and the poor skin penetration, often their use is still limited (Chang, 2009). Due to these safety concerns, the identification and isolation of new compounds from natural sources, which prevent pigment disturbances, have attracted increasing interest. Many plant extracts and compounds isolated from plant extracts have been investigated for their inhibitory effect on melanogenesis (Chaita et al., 2017), and also used as conventional skin-whitening agents (Smit et al., 2009; Chang, 2012; Khan, 2012).

The zebrafish embryo is a popular vertebrate model system for biochemical studies with high physiological and genetic similarity to mammals, therefore zebrafish embryo screens emerge as a replacement approach for animal experiments. Zebrafish have several advantages, including small size, large number of offspring in each generation, easy maintenance and handling, as well as high efficiency of drug penetration through the skin and gills. Over the past two decades, major progress has been achieved in the development of screening technologies for bioactive compounds and zebrafish have been extensively used for *in vivo* phenotype-based screens (MacRae and Peterson, 2015; Rannekamp and Peterson, 2015; Tabassum et al., 2015). Melanin pigments can be observed on the zebrafish surface allowing simple observation of the pigmentation process without any complicated experimental procedures (Logan et al., 2006; Colanesi et al., 2012). For this reason, zebrafish is already established as an *in vivo* model for evaluating the depigmenting activity of melanogenic regulatory compounds (Choi et al., 2007) and for the screening of small molecules that control pigment cell development (Colanesi et al., 2012).

In this study, we used the zebrafish model to screen *in vivo* for new natural inhibitors of melanogenesis from several endemic plant species of Greece. Among the species tested, the unpolar extract from *Crataegus pycnoloba*, a hawthorn native to the mountains of the northern and central Peloponnesus of Greece was found to significantly reduce pigmentation. Various species of *Crataegus* have been described to strengthen cardiovascular function or assist with digestion. Here, we describe the phenotype driven isolation of single active compounds belonging to the dibenzofuran family that inhibit melanogenesis and identified a molecular mechanism of how their activity is mediated.

## Materials and Methods

### General

Chemicals and reagents were from Merck (Darmstadt, Germany). Evaporation of solvents was performed on a vacuum rotary evaporator (Rotavapor R-3000r, Buchi, Switzerland). Fast Centrifugal Partition Chromatography (FCPC) was carried out on a Kromaton FCPC instrument equipped with a 1,000-mL column, adjustable rotation of 200–2,000 rpm and a preparative Laboratory Alliance pump with a pressure safety limit of 50 bar. NMR spectra in CDCl_3_ were recorded at 400 and 600 MHz (Bruker Advance III 600 MHz and DRX 400). 2D NMR experiments, including COSY, HSQC, and HMBC were performed using standard Bruker microprograms. Analytical TLC was performed on Merck Kieselgel 60 F254 or RP-8 F254 plates. Spots were visualized by UV light (254 and 365 nm) or by spraying with sulfuric vanillin. Preparative TLC was conducted on TLC Silica gel 60 F254 plates (1 mm). The selected zones were scraped and extracted with ethyl acetate to separate the corresponding compounds. Column chromatography was performed on silica gel 70–230 mesh (63–200 μm). Size exclusion chromatography was performed on Sephadex LH-20.

### Plant Material

The aerial parts of *C. pycnoloba* (Rosaceae) were collected from mountain Kyllini of Peloponnese, Greece. The plant material was identified by Dr. E. Kalpoutzakis. A voucher specimen has been deposited in the herbarium of Laboratory of Pharmacognosy and Natural Products Chemistry, Faculty of Pharmacy, University of Athens, Greece, under the number KL121.

### Extraction and Isolation

Dried pulverized aerial parts of *C. pycnoloba* (1 kg) were extracted exhaustively by maceration using initially EtOAc (3 × 2 L) and then MeOH:H_2_O 50:50 (3 × 2 L). The solvents were removed under reduced pressure to give 14.6 g of a crude EtOAc extract and 41.7 g of hydroalcoholic extract. The most active EtOAc extract (7 g) was submitted for fractionation by FCPC using a series of the biphasic systems and a stepwise-gradient elution-extrusion methodology. In more details, four biphasic systems composed of the solvents Heptane/EtOAc/EtOH/H_2_O in proportions: (a) 13/2/7/8, (b) 10/5/7/8, (c) 7/8/7/8, and (d) 4/11/7/8 were created and evaluated in test tubes by the method of Ito (2005). The distribution of the compounds between the phases of each system was evaluated by TLC analysis. This analysis showed a tiered increase of distribution of compounds in the upper phase when we moved from less to more polar phases, indicating thus a good step-gradient CPC fractionation of the mixture by using this series of biphasic systems. The solvents were thoroughly mixed in a separatory funnel at room temperature prior to use, and the two phases of each system were separated after equilibration of the mixture. The experiment started by filling the preparative CPC column (1,000 mL) with the stationary (aqueous) phase of the first system (a) at a flow rate of 10 mL/min in ascending mode. Afterward, the rotation speed of the column was increased to 850 rpm and the organic mobile phase of system (a) was pumped through the column at a flow rate of 10 mL/min for the equilibration step. When equilibrium of two phases was established, the Stationary Phase Retention (Sf), that is the ratio between the stationary phase volume and the total column (system) volume, was calculated and found to be 75%. Then 7 g of the EtOAc extract dissolved in a mixture of lower and upper phase (1:1 v/v) was injected via a 30 mL sample loop, and the experiment was started. The flow rate was established at 10 mL/min throughout the experiment, while the mobile phases were sequentially eluted using 500 mL of upper phase of each of the biphasic systems a–d. The experiment was completed by passing 800 mL of the lower phase of system d in descending mode. The fraction collector was set to collect fractions every 2 min during the experiment, resulting in 140 fractions of 20 mL each. All fractions were analyzed by TLC and were combined to give 18 fractions (CPC-Fr1 to CPC-Fr18). Fraction CPC-Fr9 (173.9 mg) was subjected to Sephadex column chromatography using EtOAc as mobile phase resulting in isolation of 7-methoxyeriobofuran (compound **1**, 5.8 mg). Furthermore, the fraction CPC-Fr10 (60.9 mg), was analyzed by Sephadex column chromatography using acetone as mobile phase resulting in the isolation of 6-hydroxy-2,3,4-trimethoxydibenzofuran (compound **2**, 8.6 mg), while preparative analysis of fractions CPC-Fr12 (25.6 mg) and CPC-Fr-11 (22.3 mg) afforded 4-demethyl-6-hydroxy-β-pyrufuran (compound **3**, 3.1 mg) and 6-hydroxy-α-pyrufuran (compound **4**, 2.9 mg), respectively. Furthermore, ursolic aldehyde (compound **5**, 5.8 mg) was isolated from a portion (20 mg) of fraction CPC-Fr8 (135 mg) by preparative TLC analysis using c-Hexane:EtOAc 80:20 as mobile phase. The known compounds (**1, 3, 4**, and **5**) were identified by means of spectral data (MS, ^1^H-NMR, ^13^C-NMR, COSY, HMQC, HMBC) and direct comparison with the respective literature data (Kemp et al., 1983; Kokuban et al., 1995a,b; Kim et al., 2005]. Compound **2** is a new natural compound and was obtained as a pale yellow powder and ^1^H and ^13^C NMR data were similar to those of its demethyl derivative 3,6-dihydroxy-2,4-dimethoxydibenzofuran (Lin et al., 2015). In more details, the ^1^H-NMR spectrum of compound **2** showed typical signals of a 1,2,3-trisubstituted benzene ring [δ 6.98 (1H, dd, *J* = 7.9, 1.0 Hz), δ 7.20 (1H, t, *J* = 7.9 Hz), and δ 7.42 (1H, dd, *J* = 7.9, 1.0 Hz)], a singlet signal (δ 7.11, 1H) arising from a pentasubstituted benzene ring, and three singlet signals corresponding to *O*-methyl groups [δ 3.97 (3H, s), δ 3.98 (3H, s) and δ 4.23 (3H, s)]. The aromatic singlet at δ 7.11, which shows long-range HMBC correlations to C-2 (δ 150.8), C-3 (δ 141.8), C-4a (δ 142.7), C-9a (δ 126.0), and C-9b (δ 120.4), was designated as H-1. The location of the methoxy groups (δ 3.97, 3.98, and 4.23) at C-2 (δ 150.8), C-3 (δ 141.8), and C-4 (δ 139.1), respectively, was confirmed by an HMBC experiment. The data indicate that the structure of **2** is 6-hydroxy-2,3,4-trimethoxydibenzofuran. ^1^H-NMR (CDCl_3_, 600 MHz), δ 7.42 (1H, dd, *J* = 7.9/1.0 Hz, H-9), 7.20 (1H, t, *J* = 7.9 Hz, H-8), 7.11 (1H, s, H-1), 6.98 (1H, dd, *J* = 7.9/1.0 Hz, H-7), 5.40 (1H, brs, OH-6), 4.23 (3H, s, CH_3_O-4), 3.98 (3H, s, CH_3_O-3), 3.97 (3H, s, CH_3_O-2). ^13^C-NMR (CDCl_3_, 600 MHz), δ 150.8 (C-2), 144.3 (C-5a), 142.7 (C-4a), 141.8 (C-3), 141.1 (C-6), 139.1 (C-4), 126.0 (C-9a), 123.8 (C-8), 120.4 (C-9b), 113.2 (C-7), 112.3 (C-9), 97.1 (C-1), 61.7 (CH_3_O-3), 61.5 (CH_3_O-4), 56.7 (CH_3_O-2).

### Zebrafish Maintenance and Breeding

Zebrafish embryos were raised under standard laboratory conditions at 28°C (Westerfield, 2000). Genetic backgrounds used were wild-type AB strains for all the screenings and the transgenic line *Tg*(*sox10*:EGFP) for monitoring *sox10* expression. Zebrafish are maintained in accordance with the European Directive 2010/63 for the protection of animals used for scientific purposes and the Recommended Guidelines for Zebrafish Husbandry Conditions^1^. The experimental protocols described in this study were carried out with zebrafish larvae up to 96 hours post fertilization (hpf) and therefore are not subject to the regulations of European animal protection guidelines.

### Compound Treatment and Phenotype Based Evaluation

For monitoring the melanogenic inhibitory activity, synchronized and dechorionated 24 hpf embryos were treated with compounds at various concentrations in 12-well plates containing 2 mL of embryo medium (0.3 g/L “Instant Ocean” Sea Salts and 0.08 g/L CaSO_4_⋅2H_2_O). Test compounds were dissolved in DMSO that was always used as vehicle control up to 0.1% (v/v) final concentration. In all experiments, 0.2 mM 1-phenyl-2-thiourea (PTU), a known TYR inhibitor, was considered as a standard positive control. Testing period varied between 24 and 48 h and the effects on the pigmentation were observed under a stereomicroscope

For the calculation of LC_50_, the OECD guidelines for Fish Embryo Acute Toxicity Test (TG 236) were used (OECD, 2013). In brief, newly fertilized zebrafish eggs were exposed to the test compounds at 3 hpf for a period of 96 h. Every 24 h lethal effects were assessed and compared to the control values. At the end of the exposure period, acute toxicity is determined and LC_50_ is calculated. Data were analyzed with SPSS statistical analysis software (Version 10.0) using Probit Analysis Statistical Method.

### Determination of Melanin Content

For determination of melanin content about 100 zebrafish embryos at 48 hpf were sonicated in cold lysis buffer (20 mM sodium phosphate (pH 6.8), 1% Triton X-100, 1 mM PMSF, 1 mM EDTA) containing protease inhibitors cocktail. An aliquot of the lysate was used to determine the protein content with a Bradford assay using BSA as the standard. The lysate was clarified by centrifugation at 10,000 × *g* for 10 min. The melanin precipitation was then resuspended with 1 mL of 1 N NaOH/20% DMSO at 95°C for 1 h. Spectrophotometric absorbance of intracellular melanin content was measured at 490 nm. The data were normalized to the total protein content of the embryo lysates.

### Tyrosinase Inhibition Assay

In our experiments, we investigated the ability of plant extracts, and isolated compounds to inhibit the oxidation of L-DOPA (L-3,4-dihydroxyphenylalanine) to dopaquinone and subsequently to dopachrome by the enzyme TYR employing a protocol from Masuda et al. (2008) with slight modifications (Chaita et al., 2017). Test samples were dissolved in DMSO to stock solutions of 10 mg/mL and were diluted in the proper concentration in phosphate buffer 1/15 M (NaH_2_PO_4_/Na_2_HPO_4_), pH 6.8; final concentrations of DMSO in the well did not exceeded 3%. In 96-well plates, 80 μL of phosphate buffer, 40 μL of sample in the same buffer and 40 μL mushroom TYR (92 units/mL), in the same buffer, were mixed. The contents of each well were incubated for 10 min at 25°C, before the addition of 40 μL of 2.5 mM L-DOPA in phosphate buffer. After incubation at 25°C for 5 min, the absorbance at 475 nm of each well was measured using a microplate reader. Blanks for every sample w/o TYR were also performed, while kojic acid was used as positive control. The percentage inhibition of the TYR activity was calculated by the following equation: [(A - B) - (C - D)]/(A - B) × 100, where A: Control (w/o sample), B: Blank (w/o sample, w/o TYR), C: Sample, D: Blank sample (w/o TYR).

### Computational Methods

The model of human AHR PAS-B domain (UniProt entry: P35869) residues 275–391 was based on the crystal structures of human HIF-1α/ARNT heterodimer (PDB ID 4H6J) (Cardoso et al., 2012) and HIF-2a/ARNT complex with an artificial ligand (PDB ID 3F1O) (Scheuermann et al., 2009). The multiple sequence alignment shown in the **Supplementary Figure S3** was obtained using Clustal Omega v1.2.1 (Sievers et al., 2011), and the homology modeling was performed with Modeller v9.10 (Fiser and Sali, 2003). Twenty models were produced and the lowest DOPE score structure was employed for validation at the SAVES server^2^, which demonstrated good statistics for a homology model (**Supplementary Figure S4**). The initial conformation of the ligands was generated from SMILES using OMEGA v2.1 (Hawkins et al., 2010), whereas input files for docking were produced with AutoDockTools v1.4.5 (Sanner, 1999). The search space was defined by a grid of 28 Å × 24 Å × 28 Å centered at 12.9, -41.8, 9.7 (*x, y, z* coordinates with respect to PDB ID 3F1O) and AutoDock Vina v1.1.2 was used for the docking calculations (Trott and Olson, 2010). Examination of the predicted complexes and preparation of the figures was carried out with VMD v1.9.2 (Humphrey et al., 1996).

### Quantitative Real-Time PCR

Total RNA from 30 hpf zebrafish embryos was extracted using TRIzol (Invitrogen) and RNA concentration and purity were determined by NanoDrop 2000c Spectrophotometer (Thermo Scientific). cDNA was synthesized using M-MLV Reverse Transcriptase (Thermo Scientific) according to the manufacturer’s instructions. PCRs were performed with a Roche cycler system (Light Cycler 96) using the KAPA SYBR FAST qPCR kit (KAPA Biosystems) and gene-specific primers.

The sequences of primers used are *β-actin* F: CGAGCTGTCTTCCATCCA, R: TCACCAACGTAGCTGTCTTCTG. *cyp1b1* F: ATCTCAGACTCCAACAGAAAAGA, R: CAAACATTAAAGCTTGTATTCGTC. *aldh3a1* F: GACAGAGTATTGGCTCTGATGAA, R: CTTTCAGGACTGTAGGAGCTATG. *utg1a* F: CTGCCACAGAATGACCTCTT, R: ATCACCATAGGCACTCCATTAC (Supplementary Table S1). The relative amounts of the different mRNAs were quantified with the ΔΔCt method (Livak and Schmittgen, 2001), while the expression of β-actin mRNA was used for normalization among samples. The fold-change ratio was calculated and expressed as mean ± SEM.

## Results

### Screening for Inhibitors of Melanogenesis From Natural Plant Extracts

**FIGURE 1:**
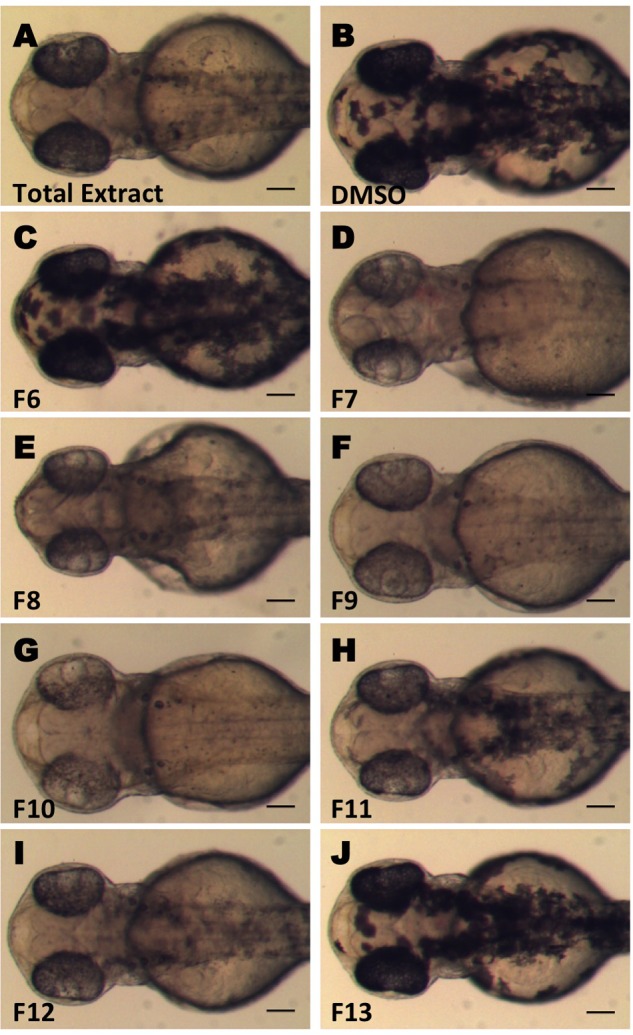
Screening for inhibitors of melanogenesis from *Crataegus pycnoloba* extracts. Crude extract **(A)** and fractions **(C–J)** were tested for anti-pigmentation ability by *in vivo* zebrafish assay. Synchronized 24 hpf embryos were treated with 0.04 mg/mL of the crude extract and 0.1 mg/mL (CPC-Fr6-8) or 0.01 mg/mL (CPC-Fr9-13) from the different fractions for 24 h. Images were obtained at 48 hpf using a stereomicroscope. Test compounds were dissolved in DMSO. **(B)** Control treated embryo. Scale bar: 100 μM.

In an effort to identify bioactive compounds, including new and potent melanogenesis inhibitors from natural sources, we performed screening using crude extracts from various endemic plant species of Greece. We used *in vivo* phenotypic zebrafish assays and we searched for extracts that inhibit melanogenesis of zebrafish embryos. Zebrafish embryos at 24 hpf were treated with extracts at concentrations ranging from 0.001 to 0.1 mg/mL and the effect on the development of melanocytes was recorded at 48 hpf (data not shown). We identified that the crude ethyl acetate extract from the aerial part of *C. pycnoloba*, a species of hawthorn native to the mountains of the northern and central Peloponnesus of Greece, decreased significantly the pigmentation of zebrafish larvae (**Figure 1A**, compare to **1B**).

### Isolation of Single Compounds With Anti-melanogenic Activity From *C. pycnoloba*

In order to identify the bioactive compounds included in the initial non-polar extract of *C. pycnoloba*, we used an activity guided subfractionation approach. Starting from the crude extract, a bioassay-guided CPC fractionation process led to the identification of six subfractions with clear anti-pigmentation activity from the total 18 (**Figures 1D–I** – fractions F7 to F12). The level of melanin reduction varied between these fractions but in all cases the effects were present at both the retina and the pigmentation pattern of the embryo. This analysis revealed that the bioactive compound(s) were present in all six consecutive fractions with a maximum concentration in fractions F9, F10. Fractions F6 and F13 (**Figures 1C,J**) as well as the fractions F1-5 and F14-18 (not shown) appeared to have no detectable effect on the pigmentation pattern.

**FIGURE 2:**
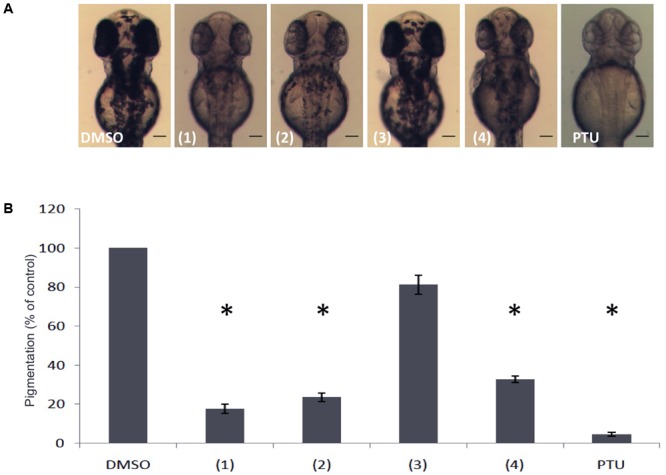
Effects of isolated compounds from the dibenzofuran family on melanin synthesis in zebrafish. **(A)** Embryos were treated from 24 to 48 hpf with 0.01 mg/mL of the compounds and the effects on the pigmentation were assessed using a stereomicroscope. **(B)** Melanin content was quantified by a photometric method. PTU was used as positive control. Results shown are the mean of three independent experiments ± SEM. ^∗^*p* < 0.001, versus DMSO control.

**FIGURE 3:**
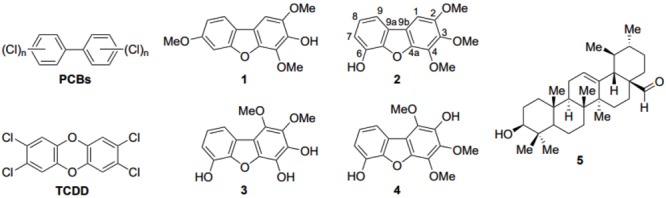
Structures of polychlorinated biphenyls (PCBs). 2,3,7,8-tetrachlorodibenzo-*p*-dioxin (TCDD) and the five compounds employed in this study. Compounds **1–4** are the dibenzo-*p*-furan derivatives isolated from the total *C. pycnoloba* extract, including the atom numbering of the newly discovered compound **2**. Compound **5** is ursolic aldehyde, isolated from the active fragments of *C. pycnoloba* but has no melanin synthesis inhibition activity.

Further purification processing using Sephadex column chromatography and preparative TLC analysis, led to the isolation of five candidate pure compounds from the above-mentioned active fractions. When tested *in vivo*, three of these compounds (**1, 2**, and **4**) were found to significantly inhibit pigmentation in the developing zebrafish larvae (**Figure 2A**). Moreover, measurement of total melanin content using whole extracts from zebrafish embryos, confirmed that treatment with these three compounds significantly decreases the amount of melanin produced after 24 h of treatment (**Figure 2B**). The level of melanogenesis inhibition varied between the three active compounds with compound **1** being the most potent in melanin reduction. Structural elucidation of all isolated compounds was performed by means of 1D and 2D NMR spectra. The chemical structure of these compounds was similar, containing a dibenzofuran unit but various substituents (**Figure 3**). Among the three active isolated compounds, compound 2 (6-hydroxy-2,3,4-trimethoxydibenzofuran) is isolated for the first time from natural sources. Interestingly, one more compound with similar structure showed no melanogenesis inhibition activity (**3, Figure 2**). As a positive control, 1-phenyl 2-thiourea (PTU), a sulfur-containing TYR inhibitor was used, which is known to inhibit pigment production in zebrafish (Elsalini and Rohr, 2003).

**FIGURE 4:**
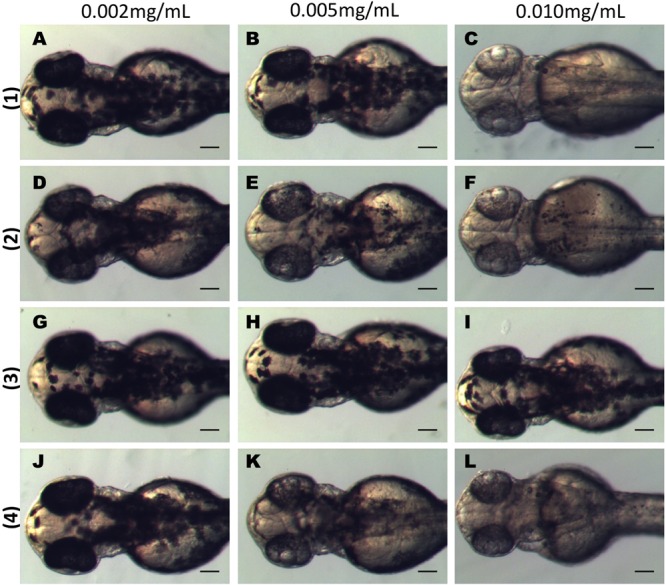
Dibenzofuran compounds inhibit melanogenesis in a dose dependent manner. Synchronized embryos were treated with test compounds at the indicated concentrations from 24 hpf for 24 h. The effects on the pigmentation of zebrafish were observed at 48 hpf under the stereomicroscope. Treatment with 0.002 mg/mL had no effect on pigmentation for any of the tested compounds **(A,D,G,J)**. Mild effect on melanogenesis was observed at 0.005 mg/mL **(B,E,K)**. At 0.01 mg/mL there was a strong inhibition of melanogenesis **(C,F,L)**. Compound **3** had no effect at any of the tested concentrations **(G–I)**. Scale bar: 100 μM.

The three active dibenzofuran compounds (**1, 2**, and **4**) were found to inhibit melanogenesis in a dose dependent manner. As shown in **Figure 4** when 24 hpf embryos were treated for 24 h with low doses (0.002 mg/mL) of the substances, the pigmentation of zebrafish was not affected. However, at increasing concentrations (0.005 mg/mL and 0.01 mg/mL) there was a remarkable inhibitory effect on the body and eye pigmentation.

Because melanin synthesis is predominantly regulated by TYR we performed an *in vitro* TYR inhibition assay that showed no inhibitory effect for the active subfractions tested in our *in vivo* assay (not shown).

### Toxicity and *in Vivo* Estimation of LC50 for the Dibenzofuran Compounds

In order to evaluate the potential cytotoxic effects of the dibenzofuran compounds we used the zebrafish embryo toxicity assay (OECD guidelines, TG 236). Treatment of the embryos started at 3 hpf and development was followed up to 96 dpf. The vehicle control treated embryos showed 100% survival rate until the end of the testing period. While **1** and **2** had similar toxicity effect toward zebrafish embryos displaying LC_50_ values of 30 μg/mL and 37 μg/mL, respectively, **4** possessed much higher toxicity resulting to an LC_50_ of 4 μg/mL. On the other hand, **3** appeared to have some toxic effects on developing embryos only at high concentrations (>100 μg/mL) (**Supplementary Figure S1**). Embryos treated with compounds (**1**) and (**2**) at the concentrations that were used for the experiments (5–10 μg/mL), never showed any lethality when we performed the OECD Fish Embryo Acute Toxicity Test and developed without any gross morphological defects or cardiac edemas, indicating that the inhibition of melanogenesis is not due to toxic effects of the compounds.

### Dibenzofuran Compounds Reversible Inhibit Melanogenesis in Zebrafish Embryos

**FIGURE 5:**
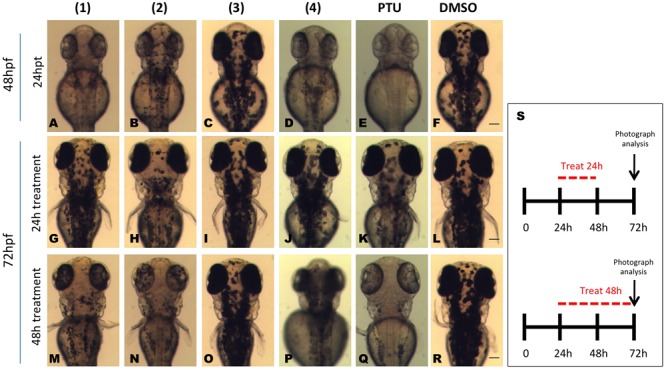
Isolated compounds from the dibenzofuran family repress reversibly *in vivo* pigmentation in zebrafish. Embryos were pretreated at 24 hpf with 0.01 mg/mL test compounds for 24 h. At 48 hpf pigmentation levels were recorded **(A–F)** and the embryos were divided into two groups. One group was washed and bathed immediately in fresh medium, whereas the other group was incubated with dibenzofuran compounds for further 24 h. At 72 hpf, the effect on melanogenesis was assessed using a stereomicroscope for both the compound treatment stopped group **(G–L)** and the embryos that were continuously treated with the dibenzofuran compounds for 48 h **(M–R)**. PTU **(E,K,Q)** and DMSO **(F,L,R)** were used as positive and negative controls, respectively. **(S)** A schematic representation for the schedule of pigmentation rescue study. Scale bar: 100 μM.

We also examined whether the effect of *in vivo* melanin inhibition from the dibenzofuran compounds was reversible. To this end, embryos at 48 hpf and 24 h post treatment were divided into two groups (**Figures 5A–F**). One group of embryos where the compounds were washed off intensively and further raised in normal embryo medium, while the other group was continuously treated with the compounds for an additional period of 24 h. The analyses of these larvae at 72 hpf revealed that embryos continuously exposed to **1, 2**, and **4** had decreased amount of pigments compared to the vehicle (DMSO) treated embryos (**Figures 5M–R**) whereas the group where the compound was washed off presented pigment recovery and the amount of melanocytes was comparable to the vehicle control (**Figures 5G–L**). However, the level of recovery of the pigmentation was not equal for all the compounds. Removal of **1** and **4** allowed the transparent embryos to almost completely restore their pigmentation pattern (**Figures 5G,J**) while removal of **2** resulted to complete retina pigmentation recovery but only partial body recuperation (**Figure 5H**).

### Treatment With Dibenzofuran Compounds Does Not Affect *sox10* Expression of Zebrafish Embryos

Sox10 is a transcription factor that initiates the melanocyte differentiation pathway and regulates a set of genes critical for pigment cell development. Mutations in *SOX10* impair pigment cell development (Hou et al., 2006). To investigate whether the identified dibenzofuran derivatives affect the differentiation of neural crest cells into melanocytes we used the zebrafish transgenic line *Tg(sox10:eGFP)* that labels the cell lineages during early neural crest development. Treatments at both 3 hpf and 24 hpf had no effect on *sox10* expression pattern up to 72 hpf. As shown in **Supplementary Figure S2**, both treated and non-treated embryos displayed similar GFP expression at the head and trunk region. *Tg(sox10:eGFP)* expression remained unchanged after treatment with **1**, as compared with the DMSO treated control, throughout the forebrain, the anterior midbrain, the otic epithelium and the branchial arches as well as in the oligodendrocytes and the Schwann cells of the trunk region.

### Molecular Modeling of AHR Complexes With Dibenzofurans

Considering that detoxification of polychlorinated dibenzo-*p*-furans is mediated though their binding to the aryl hydrocarbon receptor (AHR) (Abnet et al., 1999), we employed molecular docking calculations to investigate the potential binding of the isolated and synthesized dibenzofuran derivatives compounds **1–4** (**Figure 3**). AHR is a ligand-dependent transcription factor that binds to polychlorinated dibenzo-*p*-dioxins, including the highly potent 2,3,7,8-tetrachlorodibenzo-*p*-dioxin and polychlorinated biphenyls and responds by regulating the expression of gene programs required for their metabolism (Kewley et al., 2004; McIntosh et al., 2010). AHR and the aryl hydrocarbon receptor nuclear translocator (ARNT) belong to the basic helix-loop-helix (bHLH) family and contain a PER-ARNT-SIM (PAS) signaling domain (Taylor and Zhulin, 1999). Both the N-terminal (PAS-A) and the C-terminal (PAS-B) domains of AHR are used for heterodimerization with ARNT, still, the PAS-B domain serves as the ligand-binding domain (Denison et al., 2011). While the PAS-A domain of the mouse AHR has been recently resolved by X-ray crystallography (Wu et al., 2013), the structure of the PAS-B domain can be only inferred through its homology with other bHLH-PAS proteins, including the hypoxia-inducible factors (HIFs) (Pugh and Ratcliffe, 2003). HIF-2α was the first paradigm of the domain architecture of mammalian bHLH PAS-B to be resolved by NMR spectroscopy (Erbel et al., 2003). A crystal structure of the PAS-B domain of HIF-2α in the ligand-free form revealed a preformed internal cavity (of 290 Å^3^ volume), which albeit being solvent-inaccessible, it has been shown to accommodate artificial ligands that disrupt formation of the HIF heterodimer allosterically (Key et al., 2009; Scheuermann et al., 2009). The PAS-B domain of HIF-1α isoform that is responsible for the acute response to hypoxia has been also resolved by X-ray crystallography (Cardoso et al., 2012). Using the available structures of HIF-1α and HIF-2α PAS-B domains we prepared a model of the human AHR PAS-B domain that was employed in molecular docking of the dibenzofuran derivatives.

**FIGURE 6:**
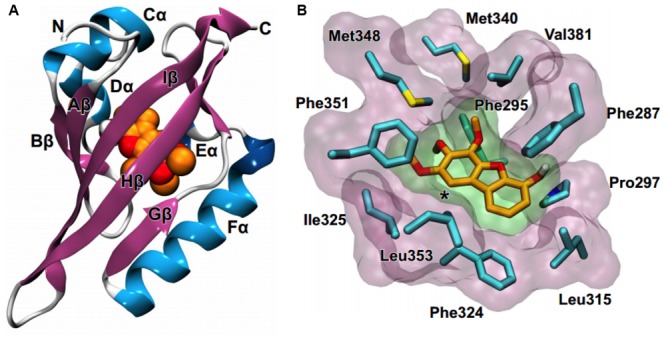
Molecular modeling suggests that compounds 1 and 2 can bind to the AHR. **(A)** Homology model of the PAS-B domain of human AHR in complex with a dibenzofuran derivative (orange C and red O spheres). **(B)** Close-up view of the internal cavity of the AHR model, illustrating a bound conformation of **2** and the surrounding hydrophobic residues (cyan C, blue N, yellow S). The asterisk indicates position-1 of the dibenzofuran ring.

The docking results showed that **1** and **2** can be accommodated inside the cavity of AHR (**Figure 6A**), displaying extended aromatic–hydrophobic interactions with the surrounding residues (**Figure 6B**). Their estimated free energies of binding suggest a binding affinity in the range of 50–100 μM. Interestingly, docking of either **3** or **4** did not produce any high-affinity bound poses inside the cavity of the AHR model. A closer examination of the AHR-**2** complex reveals that position-1 of the dibenzofuran ring, which is substituted by a methoxy group in both **3** and **4** (**Figure 3**), is at close proximity to Leu353 (**Figure 6B**). Considering the high complementarity of **1** and **2** with the internal cavity of AHR PAS-B domain, it is therefore plausible that the 1-methoxy substituted derivatives **3** and **4** cannot be properly accommodated inside the cavity due to steric effects.

### Dibenzofuran Compounds Alter the Expression of AhR Target Genes

**FIGURE 7:**
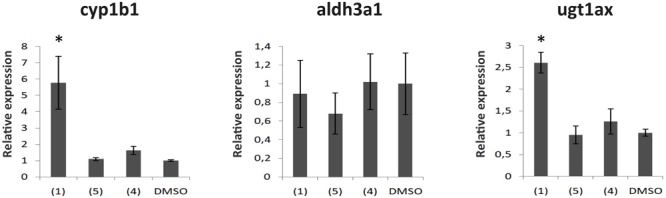
The effect of compounds from the dibenzofuran family on the mRNA expression of Ahr dependent genes. Gene expression analysis from 30 hpf zebrafish embryos treated for 6 h with 50 μg/mL of **1, 4** or **5**. Shown are mRNA levels of cyp1b1, aldh3a1, and the ugt1a superfamily, known to be downstream targets of the Ahr. mRNA expression was normalized against Actin, and the gene expression of embryos treated with DMSO (vehicle) was set as 1. Data are mean ± standard error of the mean (SEM), *n* = 3, ^∗^*P* < 0.005.

The acute effects of dibenzofurans on the expression of three AhR-dependent genes were evaluated in zebrafish. Embryos 24 hpf were treated with **1** and **4** for 6 h and the mRNA levels of *cyp1b1, aldh3a1*, and *ugt1a* were evaluated by quantitative polymerase chain reaction analysis (qPCR). A compound with no anti pigmentation effect (**5, Figure 3**) that was isolated together with the active compounds **1, 2**, and **4** from the fractions CPC-Fr6 to CPC-Fr12 fractions of the total extract of *C. pycnoloba* was also used as a negative control. This compound does not belong to the dibenzofuran family but is an ursolic aldehyde instead. Significant induction (more than five times) of cyp1b1 mRNA expression was detected only after treatment with **1**, whereas treatment with either the less active **4** or the non-active **5** resulted to cyp1b1 mRNA levels similar to the DMSO control treated embryos (**Figure 7**). *Ugt1a* PCR primers were designed to amplify together seven mRNAs from the *ugt1a* subfamily of genes. Similarly to the *cyp1b1* induction pattern, treatment of embryos with 1 significantly increased the expression levels of ugt1a genes while no differences were observed after treatment with either **4** or **5**. Interestingly, no differences were observed at the *aldh3a1* mRNA levels for embryos treated with any of the dibenzofuran compounds possibly because ALDH3A1 protein is involved in the detoxification of specific alcohol-derived acetaldehydes that might not be within the metabolites of dibenzofurans.

## Discussion

Skin pigmentation is determined by the production and distribution of melanin from melanocytes in the epidermis. Melanin protects the skin from extracellular stimuli such as solar irradiation, however, the over-accumulation of melanin can induce a number of hyperpigmentary skin conditions causing critical problems to appearance (Mort et al., 2015). Therefore, there is a need to develop depigmentation agents that are non-toxic but have a strong ability to inhibit melanogenesis. Many studies have been focused on the use of natural products in medicine and cosmetic (Antignac et al., 2011; Parveen et al., 2015). Thus, our aim was to identify new agents from natural sources that can regulate melanogenesis. In the present study, the extract from *C. pycnoloba* was discovered to have strong anti-melanogenic activity. *C. pycnoloba* is a hawthorn native to the mountains of the northern and central Peloponnesus of Greece. The plant is a shrub or rarely a small tree with white flowers and red–yellow fruits. Despite the fact that several *Crataegus* species are used in traditional Chinese medicine as digestive aids or for strengthen cardiovascular function, (Dharmananda, 2004) and the indications that *Crataegus* preparations hold significant potential as a useful remedy in the treatment of cardiovascular disease (Fong and Bauman, 2002; Tassell et al., 2010), no reports were found on the use of the endemic Greek species *C. pycnoloba*.

In the past, compounds isolated from the aerial part of another plant species from the same genus, *Crataegus azarolus*, were found to inhibit growth of B16F10 melanoma cells and to exert a potent inhibition of the melanin synthesis (Mustapha et al., 2015). They used the ethyl acetate extract obtained from the leaves of *C. azarolus* and a flavonoid component of the extract named vitexin-2″-*O*-rhamnoside, and demonstrated that both compounds have the ability to reduce the melanin content by inhibiting the TYR activity of mouse melanoma cell lines. Since melanogenesis inhibition by *C. pycnoloba* extracts has not been previously reported, we were interested in identifying the specific active compounds present within the initial crude extract. Repeated purification steps in combination with *in vivo* testing in zebrafish for all fractions and subfractions from the ethyl acetate extract of *C. pycnoloba* lead us to the identification of three compounds responsible for the anti-melanogenic activity of the crude extract. Chemically, all three compounds belong to the dibenzofuran family. They are heterocyclic organic compounds with two benzene rings fused to a central furan ring but they differ in the substitution pattern of the aromatic moieties. One of these compounds (**2**: 6-hydroxy-2,3,4-trimethoxydibenzofuran) was actually isolated for the first time in natural products. Naturally occurring dibenzofurans are mainly biosynthesized by lichens and ascomycetes, but have been also reported in the plant kingdom, marine organisms, edible mushrooms or myxomycetes. Dibenzofurans from natural sources represent a small class of secondary metabolites and have been studied for their antimicrobial, anti-inflammatory and cytotoxic effects (Millot et al., 2016).

Zebrafish is a popular vertebrate model that has been extensively used on phenotypic screens for bioactive compounds (Papakyriakou et al., 2014; MacRae and Peterson, 2015). By employing a fast and robust zebrafish screen, we identified that the dibenzofuran compounds isolated from *C. pycnoloba* exhibit strong inhibition of melanogenesis. Compounds **1 (**7-methoxyeriobofuran) and **2** (6-hydroxy-2,3,4-trimethoxydibenzofuran) are more potent since they exhibit stronger effect and lower toxicity compared to compound **4** (6-hydroxy-α-pyrufuran) while the anti-pigmenting effect was reversible and could be rescued by withdrawal of the compound.

Regulation of melanogenesis is controlled at different levels. During embryo development, melanocytes are derived from the neural crest, differentiate and subsequently migrate through the embryo to specific target positions. This migration of melanocytes is the first level of melanogenesis regulation. At the cellular level melanogenesis is regulated via the controlling formation of melanosomes that can vary in sizes, numbers and densities depending on the melanin content. At the subcellular level melanogenesis is regulating by modulating the intracellular pathways responsible for melanin synthesis that determine both the quantity and the quality of synthesized melanin (Chang, 2012). Disruption on regulation of melanogenesis is responsible for many pigmentation disorders including melanoma, the melanocyte originating cancer. The fact that melanoma is particularly aggressive and metastatic can partially be explained by the specific properties of melanocytes related to differentiation, migration and proliferation (Mort et al., 2015). Furthermore, recent studies have shown that key event in melanoma tumor initiation from a field of cancer prone melanocytes, is the reemergence of their neural crest progenitor identity (Kaufman et al., 2016). Therefore, inhibitors of melanogenesis that act at the level of melanocyte differentiation and migration could be potentially useful agents for studies related to melanoma.

Dibenzofuran derivatives isolated it the present study do not seem to interfere neither with the differentiation of neural crest melanocyte progenitors to form mature melanocytes nor with their migratory pathways. The fact that treatment of zebrafish embryos with compound **1** had no effect on the *sox10* expression pattern indicates that the early steps of neural crest differentiation have not been disrupted and the fate map of the multi potent neural crest cells has not been changed. Withdrawal of the dibenzofuran compounds from the zebrafish larvae with reduced pigmentation after compound treatment resulted on fast re-appearance of melanin along the whole embryo body. This result suggests that melanocytes migrated properly to their final target positions alongside the body but the presence of the tested compounds inhibited melanin synthesis inside the melanocytes.

Over the last few years the processes underlying melanin biosynthesis have been largely elucidated. The key enzyme on melanin production is TYR that catalyzes the tyrosine oxidation to dopaquinone. When tested *in vitro, C. pycnoloba* extracts did not inhibit significant TYR activity, despite the fact that they were able to reduce pigmentation of zebrafish larvae. Other possible mechanisms to melanosynthesis inhibition include the acceleration of TYR degradation or the inhibition of RNA transcription of melanosynthesis related genes –such as MITF or TYR – but these possibilities have to be examined further.

Here, we explored the activity of Ahr, a well-studied receptor of similar, albeit chemically synthesized polychlorinated dibenzo-*p*-furans, through which their toxic and biochemical effects are mediated (Li et al., 2011). To this end, we employed an integrated molecular docking approach to investigate the potential binding interactions between naturally occurring *C. pycnoloba* dibenzofurans and AHR. Our calculations revealed that the more active compounds **1** and **2** can be accommodated inside the cavity of AHR, displaying extended aromatic–hydrophobic interactions with the surrounding residues. Docking of either the non-active compound **3** or the less active **4** did not produce any high-affinity bound pose inside the cavity of the AHR. Therefore, it is plausible that substitution at position-1 of their scaffold is mainly responsible for diminishing the binding of **3** and **4** with respect to **1** and **2**. In accordance to the molecular docking results, treatment of zebrafish embryos with **1** and not with **4** or other non-active compounds induced the expression of CYP1B1 and UGT1A, known targets of the AHR. Both of these proteins are involved in metabolic pathways related to xenobiotic exposure. CYP1B1 is an oxidoreductase, and belongs to the cytochrome P450 superfamily of enzymes (Faiq et al., 2014) while UGT1A is an UDP-glucuronosyltransferase that is involved in conjugation reactions during xenobiotic metabolism (Strassburg et al., 2008).

During the last few years there is accumulating evidence for the implication of AHR in melanogenesis. Activation of AHR by tetrachlorodibenzo-*p*-dioxin (TCDD) resulted in induction of TYR activity and elevated melanin content in human melanocytes (Luecke et al., 2010). Moreover, it has been shown that UVB induced tanning on mice is mediated by AHR activation. However, in the same work, ligand-induced activation of AHR in primary melanocyte cultures did not drive *de novo* transcription of melanogenic enzymes (including TYR), TYR activation and melanin production (Jux et al., 2011). The authors of this article suggested that AHR signaling is involved in the homeostasis and/or differentiation of melanocytes and melanocyte precursors, but not in the direct induction of genes related to melanin synthesis. More recently it was found that benzo[a]pyrene that is known to activate AHR and induce CYP1A1 represses melanogenesis in B16F10 mouse melanoma cells (Joo et al., 2015). All these data provide some evidence that AHR might be implicated in the melanogenesis process, although its exact role remains elusive. Regarding the substituted dibenzofuran compounds isolated in this study, further studies are required to investigate whether the observed melanogenesis inhibition by **1** and **2** is mediated through binding to Ahr, or if triggering of the Ahr signaling by such compounds is related to their metabolism and biotransformation.

In summary, we employed zebrafish as a tool for screening for new natural compounds that inhibit melanogenesis. We identified for the first time two compounds from the Greek endemic plant *C. pycnoloba*, which reversibly reduce pigmentation in zebrafish embryos. These compounds belong to the dibenzofuran family and display low toxicity and high efficiency on pigment inhibition. Our results suggest that these compounds do not interfere with melanocyte differentiation or migration, but rather regulate melanin synthesis within the melanocytes without significantly lowering the TYR activity. Molecular modeling suggests that they can bind to AHR and qPCR experiments demonstrated that they can activate downstream effector genes of AHR. Further studies to elucidate the exact molecular mechanisms of these active dibenzofurans on the control of melanogenesis are warranted.

## Author Contributions

DB, A-LS, and NA conceived and designed the experiments. NA, MT, and ApA isolated and provided the natural product extracts. AdA designed and conducted the zebrafish experiments. AP performed the molecular modeling analysis. DB, AdA, A-LS, AP, and NA wrote and revised the manuscript.

## Conflict of Interest Statement

The authors declare that the research was conducted in the absence of any commercial or financial relationships that could be construed as a potential conflict of interest.

## Footnotes

1 https://www.eufishbiomed.kit.edu/59.php

2 http://services.mbi.ucla.edu/SAVES

## Supplementary Material

FIGURE S1 Lethal concentrations for the compounds of the dibenzofuran family. Zebrafish embryos were incubated with compounds **1, 2, 3**, and **4** from 3 hpf up to 96 hpf. Survival as scored and LC_50_ values were calculated based on the OECD guidelines for Fish Embryo Acute Toxicity Test (TG 236). Data are mean ± standard error of the mean (SEM).

FIGURE S2 Treatment with the dibenzofuran derivatives does not affect the expression of *sox10* in zebrafish embryos. Lateral views of transgenic embryos 72 hpf from line *Tg(sox10:GFP)* treated from 3 hpf with DMSO **(A,B)** or the compound 1 **(C,D)**. Both treated and non-treated embryos show extensive GFP expression throughout forebrain **(A)**, anterior midbrain **(B)**, otic epithelium **(C)**, and branchial arches **(D)** while in lateral views of the trunk there is increased GFP expression in oligodendrocytes (arrow) and Schwann cells (arrowhead).

FIGURE S3 Multiple sequence alignment of the PAS-B domains. Human AHR (275–391), HIF-1α (238–347), and HIF-2α (234–349) employed in the homology modeling. The sequence identity of AHR PAS-B domain with the corresponding residues of HIF-1α and HIF-2α is 26.7% and 25.4%, respectively.

FIGURE S4 Ramachandran plot from a PROCHECK analysis of the PAS-B domain residues 275–391 of the human AHR model used in the docking calculations.

TABLE S1 Sequences of primers used in real-time PCR.
