# Supplementary figures and images for: Identification of Novel Melanin Synthesis Inhibitors From Crataegus pycnoloba Using an in Vivo Zebrafish Phenotypic Assay
Source: Front Pharmacol. 2018 Mar 26;9:265. doi: 10.3389/fphar.2018.00265 (PMC5879087; doi:10.3389/fphar.2018.00265)

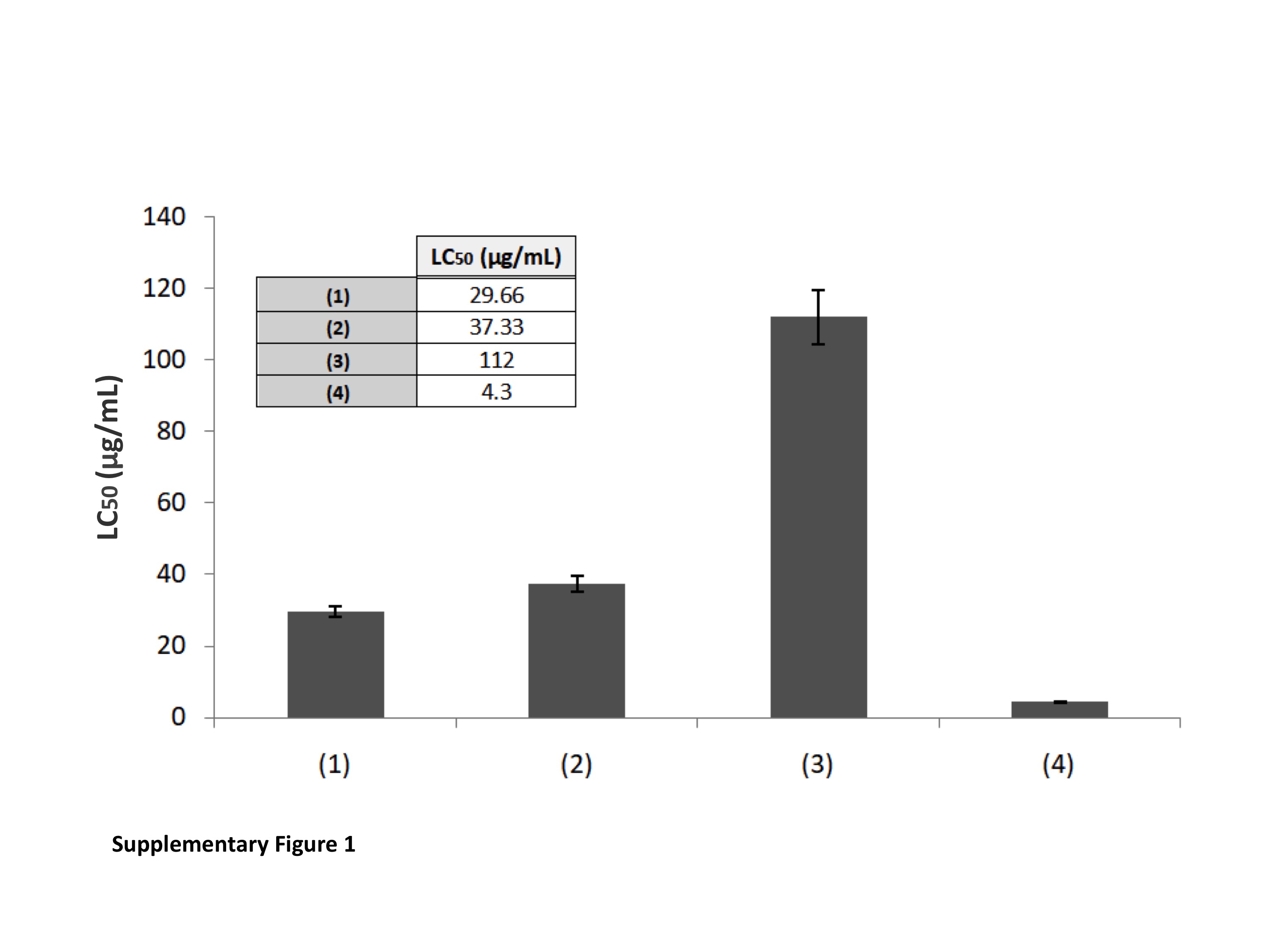

Supplement: FIGURE S1 — Lethal concentrations for the compounds of the dibenzofuran family. Zebrafish embryos were incubated with compounds 1, 2, 3, and 4 from 3 hpf up to 96 hpf. Survival as scored and LC50 values were calculated based on the OECD guidelines for Fish Embryo Acute Toxicity Test (TG 236). Data are mean ± standard error of the mean (SEM). [file Image_1.JPEG]

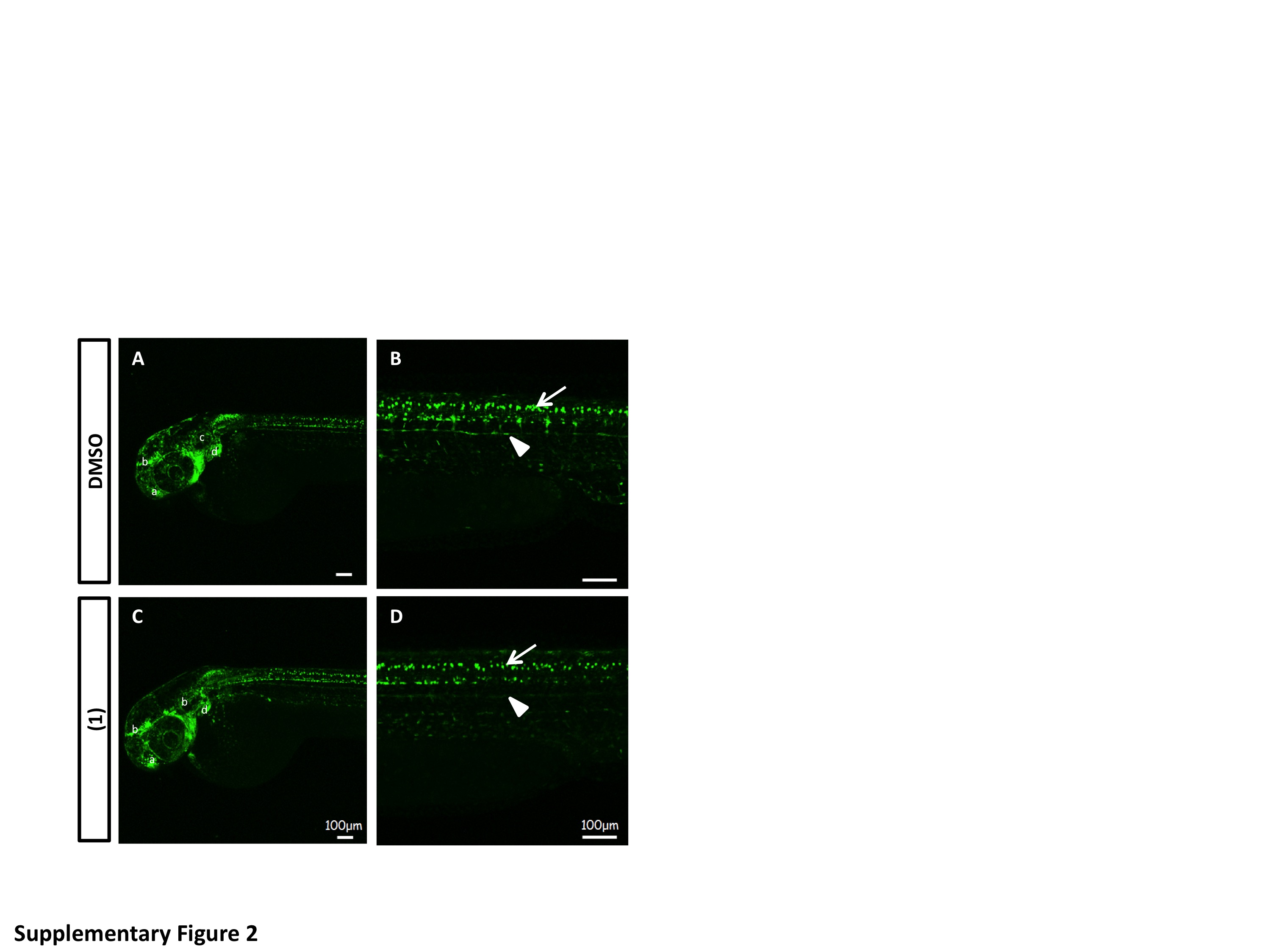

Supplement: FIGURE S2 — Treatment with the dibenzofuran derivatives does not affect the expression of sox10 in zebrafish embryos. Lateral views of transgenic embryos 72 hpf from line Tg(sox10:GFP) treated from 3 hpf with DMSO (A,B) or the compound 1 (C,D). Both treated and non-treated embryos show extensive GFP expression throughout forebrain (A), anterior midbrain (B), otic epithelium (C), and branchial arches (D) while in lateral views of the trunk there is increased GFP expression in oligodendrocytes (arrow) and Schwann cells (arrowhead). [file Image_2.JPEG]

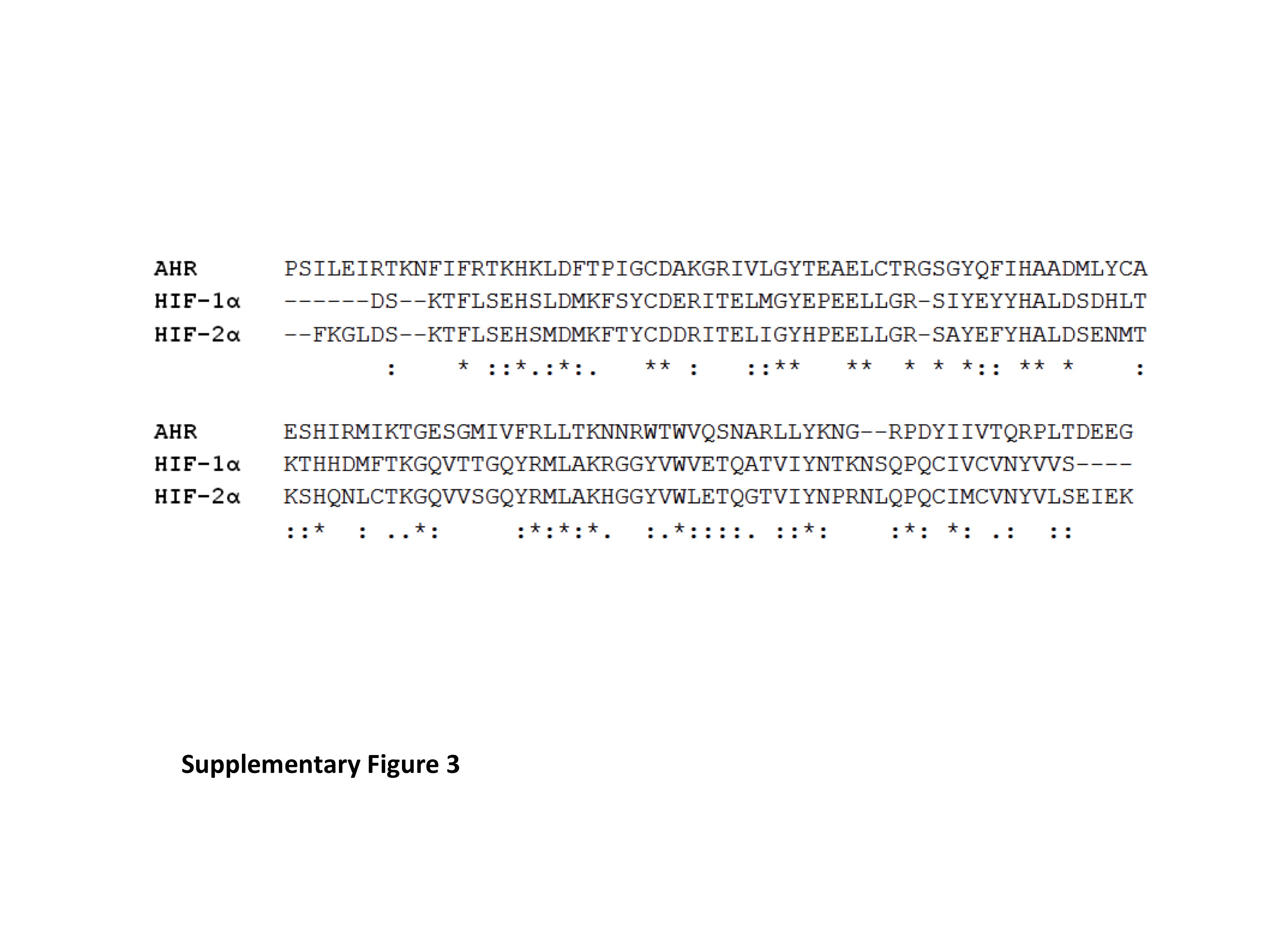

Supplement: FIGURE S3 — Multiple sequence alignment of the PAS-B domains. Human AHR (275–391), HIF-1α (238–347), and HIF-2α (234–349) employed in the homology modeling. The sequence identity of AHR PAS-B domain with the corresponding residues of HIF-1α and HIF-2α is 26.7% and 25.4%, respectively. [file Image_3.JPEG]

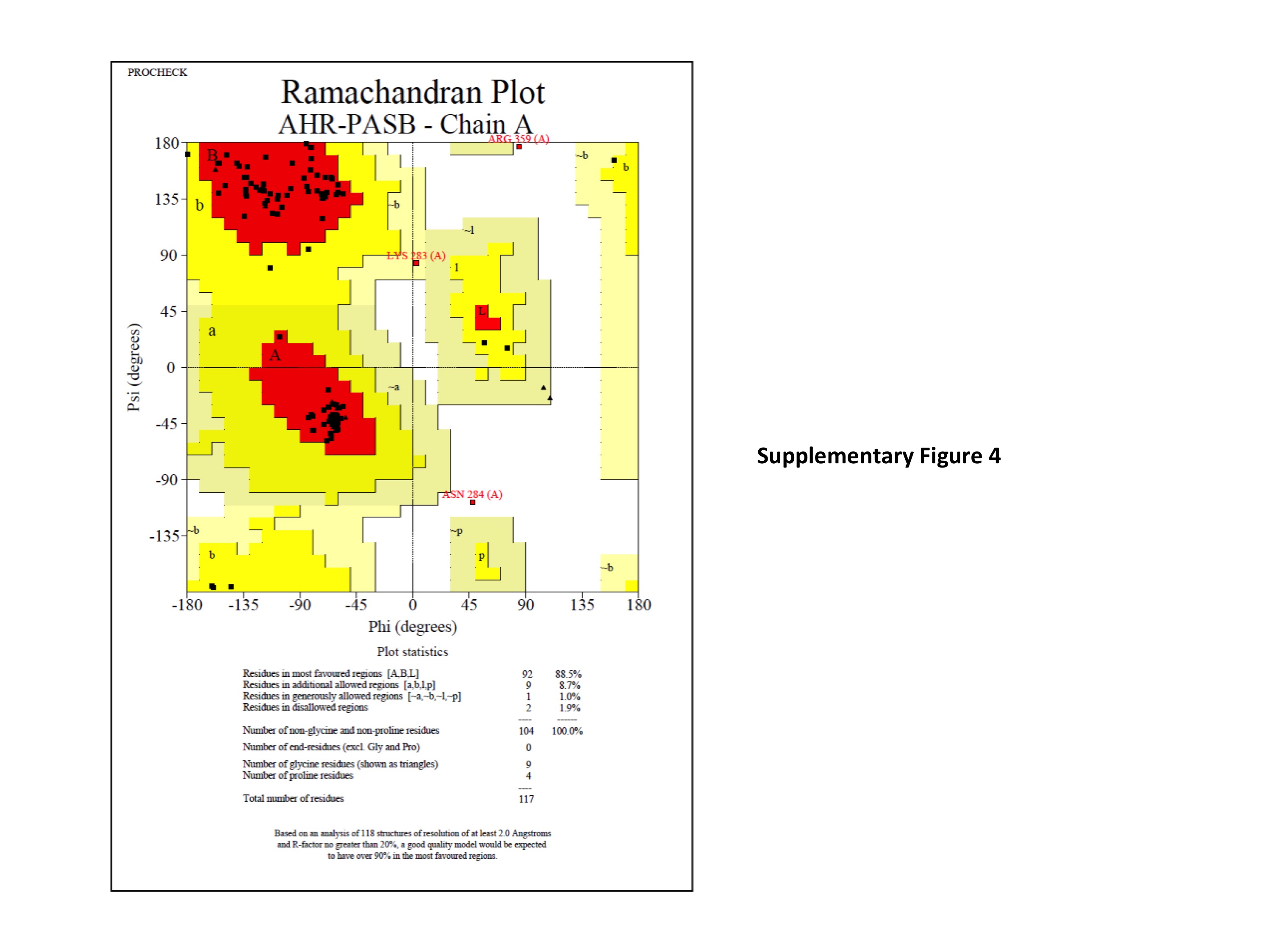

Supplement: FIGURE S4 — Ramachandran plot from a PROCHECK analysis of the PAS-B domain residues 275–391 of the human AHR model used in the docking calculations. [file Image_4.JPEG]

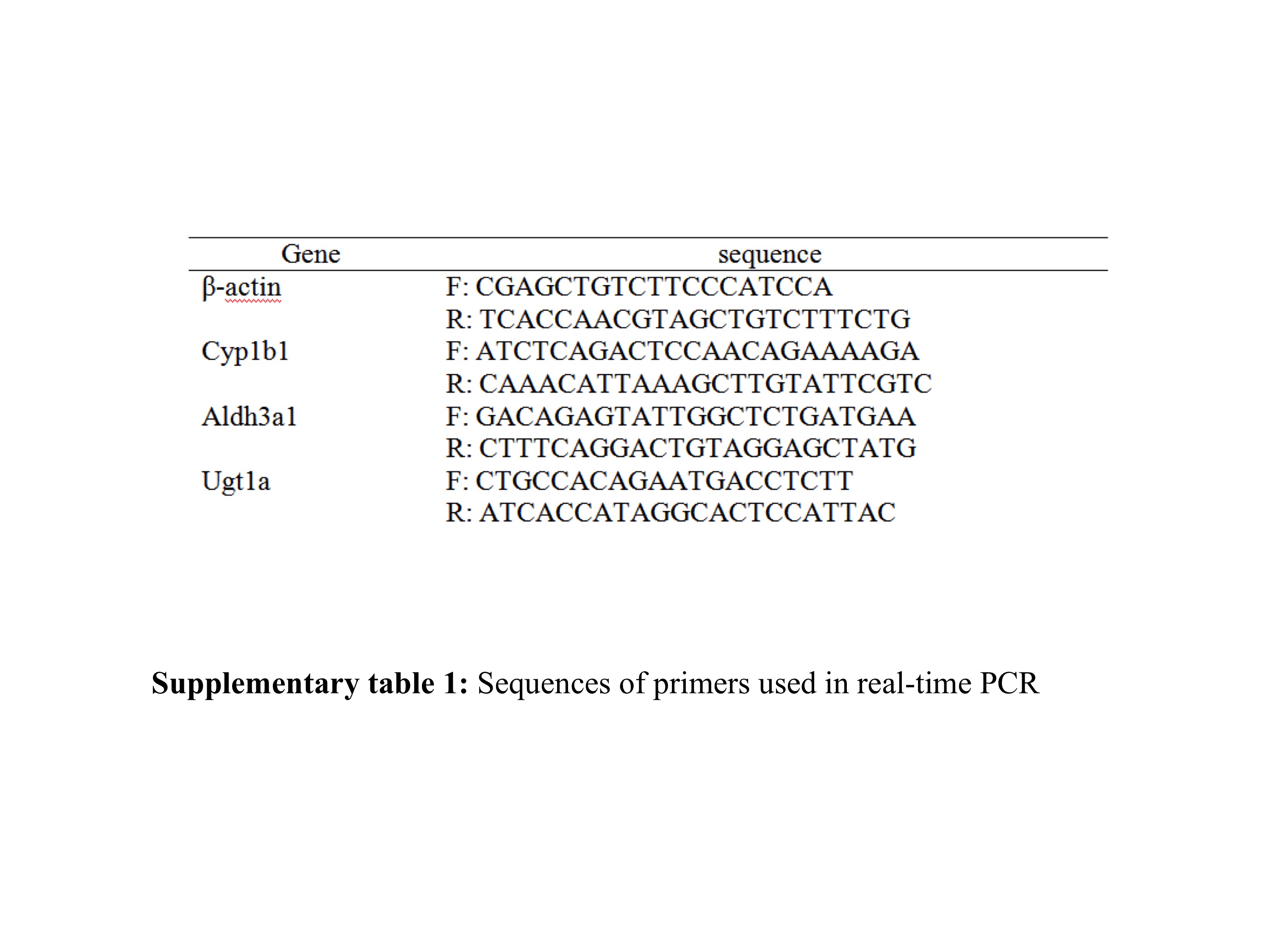

Supplement: TABLE S1 — Sequences of primers used in real-time PCR. [file Image_5.JPEG]
